# Labial adhesions due to vulvovaginal lichen planus suspected to be caused by angiotensin II receptor blocker

**DOI:** 10.1002/iju5.12815

**Published:** 2024-12-18

**Authors:** Yugo Sawada, Yasuhide Kitagawa, Atsushi Tanaka, Shunsuke Harada, Masayoshi Jimmy Nomura

**Affiliations:** ^1^ Urogynecology Center Kameda Medical Center Kamogawa Chiba Japan; ^2^ Department of Urology Tokyo Women's Medical University Tokyo Japan; ^3^ Department of Urology Komatsu Municipal Hospital Komatsu Kanazawa Japan; ^4^ Department of Dermatology Kameda Medical Center Kamogawa Chiba Japan; ^5^ Department of Pathology Kameda Medical Center Kamogawa Chiba Japan

**Keywords:** adverse event, labial adhesions, lichen planus, lichenoid drug eruption, vulvodynia

## Abstract

**Introduction:**

Lichenoid drug eruption, induced by gold or cardiovascular drugs, is one of the causes of lichen planus, and vulvovaginal erosive lichen planus can cause labial adhesions. However, few studies have focused on drugs as a cause of labial adhesions.

**Case presentation:**

We encountered a 78‐year‐old woman with labial adhesions, vulvodynia, and itching of the vulva. The cause was thought to be lichenoid drug eruption from an angiotensin II receptor blocker. After discontinuation of the drug, vulvodynia and pruritus resolved quickly, the pathology showed improvement, and labial adhesions did not recur after detachment.

**Conclusion:**

An angiotensin II receptor blocker was a suspected cause of vulvovaginal erosive lichen planus, which causes labial adhesions. Physicians should recognize the possible cause of labial adhesions secondary to lichenoid drug eruption.

**Keynote message**

Lichen planus, known to be caused by drugs, is a well‐known cause of labial adhesions. However, reports of labial adhesions related to lichenoid drug eruptions (drug‐induced lichen planus) are rare. Physicians should be aware of the possibility that drugs may cause labial adhesions.

**Abbreviations & Acronyms**
ARB: angiotensin II receptor blocker
DLST: drug‐induced lymphocyte stimulation test
OABSS: overactive bladder symptom score
PVR: post‐void residual urine volume
UFM: uroflowmetry

## Introduction

Labial adhesions are generally attributed to estrogen deficiency and are primarily observed in prepubertal girls and postmenopausal women. Other possible causes include localized chronic inflammation due to poor hygiene, recurrent urinary tract infections, and skin diseases such as lichen sclerosis and lichen planus.^1^ It has been reported that 42–59% of patients with vulvovaginal lichen planus have vaginal stenosis and labial adhesions.^2^, ^3^ Lichenoid drug eruption, for which gold and cardiovascular drugs are known to be causative agents,^4^, ^5^ is one of the causes of lichen planus. However, only few studies have reported on the relationship between labial adhesions and lichenoid drug eruption.

Herein, we report the case of a patient with labial adhesions due to vulvovaginal lichen planus, which was suspected to be caused by an ARB.

## Case presentation

**Fig. 1:**
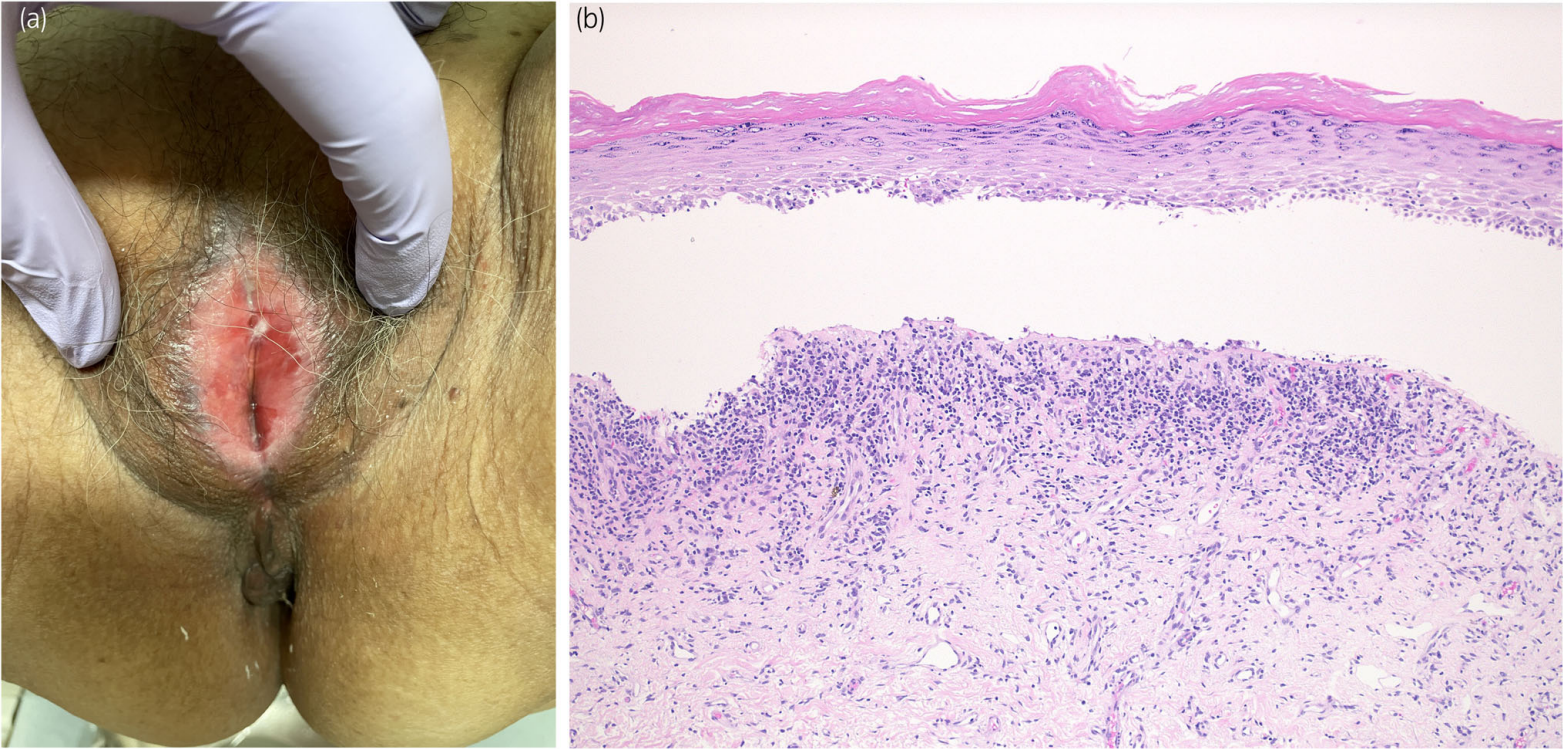
Before discontinuation of ARB. (a) Photograph of the vulva at the initial examination. Labial adhesions are observed at the 0 and 6 o'clock positions at the vaginal introitus, resulting in vaginal introitus stenosis. Mucosal erythema and lace‐like hyperkeratosis are observed mainly in the vaginal introitus. (b) Skin biopsy at the time of labia detachment showing hyperkeratosis, mildly atrophic epidermis with flattened basal keratinocytes, and a band‐like infiltrate of chronic inflammatory cells involving the subepidermal dermis. Civatte bodies are also observed in the suprabasal layers (Hematoxylin and eosin; total magnification ×100).

**Fig. 2:**
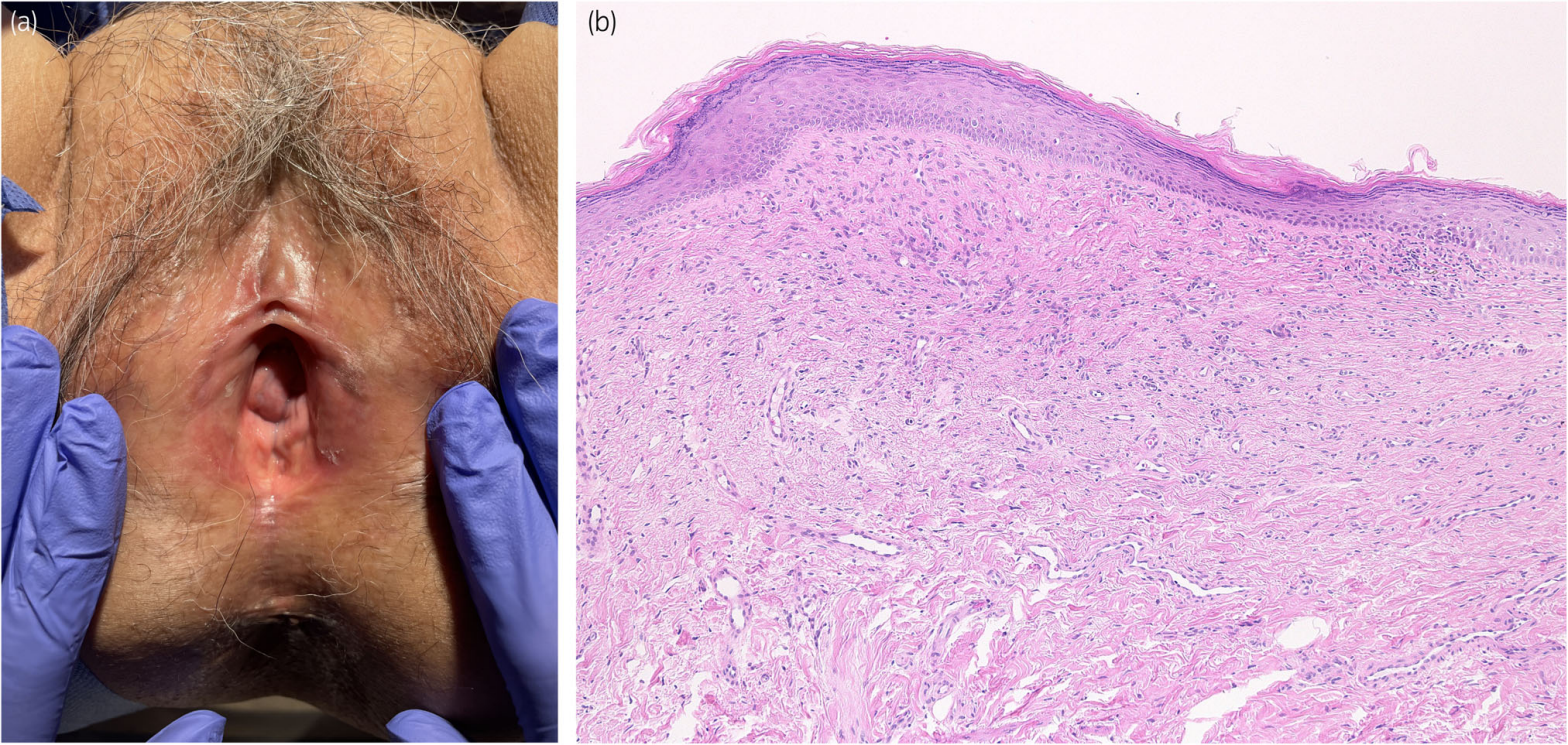
Two months after discontinuation of ARB. (a) Photograph of the vulva showing that the redness of the vulvar mucosa has improved due to lessened vulvar pain and itching. (b) Skin biopsy showing hyperkeratosis and mild chronic inflammatory cell infiltration in the subepidermal dermis (Hematoxylin and eosin; total magnification ×100).

A 78‐year‐old woman was referred to our department with complaints of vulvar pain, itching, and decreased urinary output for >5 years. She had been treated with a steroid ointment by her previous physician, but it was ineffective. Her OABSS was 6 (Q1: 1, Q2: 1, Q3: 3, and Q4: 1). Physical examination of the vulva revealed labial adhesions, with erythema and mucosal erosion (Fig. 1a). A preoperative UFM could not be evaluated due to severe dysuria, and the PVR was 26 mL. She was taking candesartan cilexetil, pitavastatin calcium hydrate, alfacalcidol, and mirabegron for hypertension, hyperlipidemia, osteoporosis, and overactive bladder. She had a history of allergic reactions to procaine hydrochloride, omeprazole, and levofloxacin. Blunt separation, sharp dissection, and a skin biopsy with a 3‐mm punch were performed under lumbar anesthesia. Pathologic examination revealed a vulvovaginal lichen planus (Fig. 1b). After labia detachment, the difficulty in urination improved. One month after surgery, her OABSS was 0 (Q1: 0, Q2: 0, Q3: 0, and Q4: 0), and UFM findings were within the normal range (voided volume: 216 mL, maximum flow rate: 18.9 mL/s, average flow rate: 12.7 mL/s, PVR: 0 mL). Clobetasol propionate ointment was prescribed postoperatively; however, the patient's symptoms of itching and pain in the vulva did not subside, and the labial adhesions tended to recur. Candesartan cilexetil was considered as a possible cause of the lichenoid drug eruption and was discontinued 3 months after debridement. The patient's symptoms and redness of the vulvar mucosa improved within 1 week. Skin biopsy performed 2 months after discontinuation of the drug showed improvement of the lichen planus (Fig. 2a,b). There was no recurrence of labial adhesions for 4 months after discontinuation of candesartan cilexetil. A DLST was performed twice for the medications she was taking at the time of the initial diagnosis, including candesartan cilexetil, and all drugs tested negative on both occasions. A challenge test with candesartan cilexetil was offered to the patient; however, she did not accept it.

## Discussion

Lichen planus is a recognized cause of labial adhesions,^2^, ^3^ and multiple clinicopathologic diagnostic criteria have been proposed for vulvar erosive lichen planus.^6^, ^7^ According to those endorsed by the International Society for Vulvovaginal Diseases, the criteria are as follows: (i) a well‐demarcated, glazed red macule or patch in the labia minora, vestibule, and/or vagina; (ii) disease affecting hairless skin, mucocutaneous junction, and/or nonkeratinized squamous epithelium; (iii) evidence of basal layer damage, categorized as degenerative or regenerative; (iv) a closely applied band‐like lymphocytic infiltrate; and (v) absent subepithelial sclerosis.^6^ These diagnostic criteria for the pathologic findings of erosive lichen planus were compatible with the findings in our patient.

Several instances of ARB‐induced lichenoid drug eruption have already been reported at various sites, including vulvovaginal lesions (Table 1).^8^, ^9^, ^10^ These patients showed improvement of symptoms within a few weeks after discontinuation of treatment with the causative agents, and the reported clinical courses were similar to those in our patient, whose labial adhesions may be associated with lichenoid drug eruption caused by ARB.

**Table 1:** Case reports of lichenoid drug eruption due to ARB

Author	Age at lichenoid drug eruption onset	Sex	Drug	Site of eruption	Treatment	After treatment	DLST	Patch test
Pfab *et al*. (2006)	75	Female	Losartan/hydrochlorothiazide	The whole body, sparing the facial region and oral cavity	Changing to other antihypertensive drugs and topical steroid ointment treatment	Improved	None	Positive only for patches of combined drugs, negative for patches of each single drug
Ruiz‐Villaverde and Galan‐Gutierrez (2011)	66	Male	Eprosartan/hydrochlorothiazide	On the chest, both forearms, back, and thighs	Discontinued the drug and administrated 1 mg/kg/day of prednisone	Improved	None	Positive only for patches of combined drugs, negative for patches of each single drug
Gencoglan *et al*. (2009)	46	Female	Valsartan	On the posterolateral chest. The oral and genital mucosae, nails, and scalp were not involved.	Changing to another antihypertensive drug and topical steroid ointment treatment	Improved	None	None
Gencoglan *et al*. (2009)	57	Male	Valsartan	On the left neck and chin. The nails, scalp, oral and genital mucosa were intact	Changing to another antihypertensive drug and topical steroid ointment treatment	Improved	None	None
This case	78	Female	Candesartan	On the vulva	Discontinued the drug and topical steroid ointment treatment	Improved	Negative	None

Although the etiopathogenesis of lichen planus is not fully understood, it is thought to be caused by a T‐cell‐dominant autoimmune response against keratinocytes in the basal cell layer.^11^ In addition, lichen planus has been associated with exogenous agents such as viruses, contact allergens, and drugs.^11^ These exogenous antigens have been reported to play a role in priming cytotoxic T cells, which then damage basal keratinocytes, leading to the subsequent lichen planus development.^12^ One possible explanation for the wide variety of etiologies of lichenoid eruption is that the pathogenesis results from a delayed allergic reaction to epitopes of exogenous substances. In lichenoid eruptions caused by ARBs, including the one in our patient, DLST and patch tests for a single drug were negative, but those for a fixed‐dose, combination of drugs were positive.^8^, ^9^ This can be explained as a compound‐induced allergy. Moreover, a strong covalent bond with the thiol group of cysteine in the drug structure may lead to hapten formation and induce a lichenoid drug eruption,^13^ suggesting the complexity of the etiologic interpretation. Our case may not result from candesartan alone, as the combination of other etiologies with candesartan might be involved.

For labial adhesions, debridement is performed in patients with severe presentations who do not respond to the commonly used estrogen and topical steroids or have dysuria; however, reattachment was observed in 26–39% of patients.^14^, ^15^ A good clinical course, including a follow‐up skin biopsy in our patient, might reveal that labial adhesions caused by lichen planus can be cured without recurrence by discontinuation of the causative agent and removal of the labial adhesions. Physicians, including gynecologists and urologists, should recognize the possibility that ARBs can cause labial adhesions.

Labial adhesions caused by lichen planus can be improved by discontinuing treatment with the causative agent and performing labial fusion debridement. Further clarification of the mechanism of vulvar lichenoid drug eruptions is desirable.

## Author contributions

Yugo Sawada: Conceptualization; writing – original draft; project administration; resources. Yasuhide Kitagawa: Supervision; writing – review and editing. Atsushi Tanaka: Writing – review and editing; supervision. Shunsuke Harada: Writing – review and editing; resources. Masayoshi Jimmy Nomura: Writing – review and editing; supervision.

## Conflict of interest

The authors declare no conflict of interest.

## Approval of the research protocol by an Institutional Reviewer Board

The study design was approved by the appropriate ethics review board.

## Informed consent

We have obtained written consent from the patient for the potential publication of her case and photos.

## Registry and the Registration No. of the study/trial

18‐191.
